# Alcohol-related changes in the intestinal microbiome influence neutrophil infiltration, inflammation and steatosis in early alcoholic hepatitis in mice

**DOI:** 10.1371/journal.pone.0174544

**Published:** 2017-03-28

**Authors:** Patrick P. Lowe, Benedek Gyongyosi, Abhishek Satishchandran, Arvin Iracheta-Vellve, Aditya Ambade, Karen Kodys, Donna Catalano, Doyle V. Ward, Gyongyi Szabo

**Affiliations:** 1 Department of Medicine, University of Massachusetts Medical School, Worcester, Massachusetts, United States of America; 2 Center for Microbiome Research, University of Massachusetts Medical School, Worcester, Massachusetts, United States of America; 3 Department of Microbiology and Physiological Systems, University of Massachusetts Medical School, Worcester, Massachusetts, United States of America; Texas A&M University, UNITED STATES

## Abstract

**Background:**

Alcohol-induced intestinal dysbiosis disrupts homeostatic gut-liver axis function and is essential in the development of alcoholic liver disease. Here, we investigate changes in enteric microbiome composition in a model of early alcoholic steatohepatitis and dissect the pathogenic role of intestinal microbes in alcohol-induced liver pathology.

**Materials and methods:**

Wild type mice received a 10-day diet that was either 5% alcohol-containing or an isocaloric control diet plus a single binge. 16S rDNA sequencing defined the bacterial communities in the cecum of alcohol- and pair-fed animals. Some mice were treated with an antibiotic cocktail prior to and throughout alcohol feeding. Liver neutrophils, cytokines and steatosis were evaluated.

**Results:**

Acute-on-chronic alcohol administration induced shifts in various bacterial phyla in the cecum, including increased Actinobacteria and a reduction in Verrucomicrobia driven entirely by a reduction in the genus *Akkermansia*. Antibiotic treatment reduced the gut bacterial load and circulating bacterial wall component lipopolysaccharide (LPS). We found that bacterial load suppression prevented alcohol-related increases in the number of myeloperoxidase- (MPO) positive infiltrating neutrophils in the liver. Expression of liver mRNA tumor necrosis factor alpha (*Tnfα*), C-X-C motif chemokine ligand 1 (*Cxcl1*) and circulating protein monocyte chemoattractant protein-1 (MCP-1) were also reduced in antibiotic-treated alcohol-fed mice. Alcohol-induced hepatic steatosis measured by Oil-Red O staining was significantly reduced in antibiotic treated mice. Genes regulating lipid production and storage were also altered by alcohol and antibiotic treatment. Interestingly, antibiotic treatment did not protect from alcohol-induced increases in serum aminotransferases (ALT/AST).

**Conclusions:**

Our data indicate that acute-on-chronic alcohol feeding alters the microflora at multiple taxonomic levels and identifies loss of *Akkermansia* as an early marker of alcohol-induced gut dysbiosis. We conclude that gut microbes influence liver inflammation, neutrophil infiltration and liver steatosis following alcohol consumption and these data further emphasize the role of the gut-liver axis in early alcoholic liver disease.

## Introduction

The National Institute on Alcohol Abuse and Alcoholism reports that 17 million adults in the US have an alcohol use disorder, approximately 1.4 million adults receive treatment for this disorder, and close to 88,000 people die each year of alcohol related causes [1]. Chronic excessive alcohol use can lead to liver cirrhosis and alcoholic hepatitis, a condition with high mortality. The trigger(s) for acute alcoholic hepatitis are yet to be identified; however, the importance of the gut-liver axis is increasingly recognized in alcoholic liver disease and in decompensation in other chronic liver diseases.

Animal and human studies have shown that alcohol consumption causes “leaky gut,” translocation of bacteria and microbial compounds across the intestinal basement membrane into the portal and systemic circulations [1, 2]. Recently, we have shown that an acute dose of alcohol increases circulating markers of bacterial-product translocation across the gut barrier even in healthy individuals [3]. Additionally, alcohol consumption leads to intestinal bacterial dysbiosis and bacterial overgrowth in the small intestine in humans as well as in mouse models of alcohol consumption [4, 5]. The liver is a primary site for removal of bacteria and bacterial products [6] that translocate across the intestinal barrier and into the portal circulation [7]. Once in the liver, bacterial products such as lipopolysaccharide (LPS) activate TLR-4 signaling pathways and initiate an innate immune response thereby augmenting the damage caused by alcohol’s primary insult on hepatocytes [8, 9]. The characteristic histological feature of inflammation in acute alcoholic hepatitis is the presence of neutrophils [10]. While the exact role of neutrophils is yet to be defined in alcoholic liver disease, the clinical outcome in patients correlates with the presence of neutrophils in the liver in alcoholic hepatitis [11].

Various animal models replicate aspects of alcoholic liver disease in mice. Among them, the acute-on-chronic feeding model utilizes a liquid diet containing 5% ethanol by volume for ten days followed by an acute binge [12]. This model is emphasized for its replication of neutrophil infiltration in the liver, mimicking acute steatohepatitis in human patients [13], but it is not known if alcohol-related changes in the intestine, such as dysbiosis, are replicated in this new model.

We hypothesized that enteric bacterial products play an important role in acute alcoholic hepatitis as demonstrated in the acute-on-chronic model of steatohepatitis in mice. Sequencing of enteric bacteria revealed minimal shifts in composition after this short feeding model and identified changes in the genus *Akkermansia* as an early indicator of alcohol-induced dysbiosis. We further postulated that the gut-liver axis particularly effects the infiltration of neutrophils. We show that suppression of intestinal bacterial load with antibiotics reduced neutrophil infiltration and the inflammatory response in the liver. We also report that, independent from the presence of bacterial products, liver damage may still occur. Together, these data offer critical information regarding the gut-liver axis and its role in alcoholic liver disease and in a frequently used alcohol feeding model.

## Methods

### Mice and alcohol feeding

All animals were cared for in strict accordance with the approved Institutional Animal Care and Use Committee protocol specific to the procedures described in this study at the University of Massachusetts Medical School (Protocol #A-1154-14; G.S.). Wild-type C57BL/6 6- to 8-weeks-old female mice were purchased from Jackson Laboratories and were cohoused in the University of Massachusetts Medical School Animal Medicine Facility for one week prior to the start of the experiment at which time they were doubly housed. Mice were treated with an alcohol feeding model described by Bertola et al. [12]. Briefly, all mice were fed the Lieber-DeCarli pair-fed diet for five days to become acclimated to a liquid diet. Some mice were then switched to the Lieber-DeCarli ethanol diet containing 5% ethanol and maltose dextrin (to control for caloric intake). Pair-fed mice were calorie matched with the ethanol-fed mice. On the tenth day, mice were gavaged between midnight and 2am with either ethanol (5 g/kg body weight (BW)) or isocaloric maltose dextrin. Mice were cheek bled, anesthetized under ketamine (100mg/kg BW) and xylazine (10mg/kg BW) and then euthanized by exsanguination and bilateral pneumothorax at 9h post-gavage (9-11am). Throughout the experiments, animals were monitored at least twice daily (in the morning and the evening) by our laboratory as well as regular wellness checks by University of Massachusetts Medical School veterinary technicians and all efforts were made to minimize suffering. Following oral gavage, animals were monitored for respiratory distress, evidence of accidental airway gavage. Four animals out of 50 exhibited respiratory distress following an oral gavage (of either antibiotics or ethanol) and were euthanized according to our protocol.

### 16S rDNA sequencing

Cecal contents were collected from some animals and frozen at -80°C. DNA was extracted using Stool DNA Extraction Kit (Qiagen) according to the manufacturer’s instructions. To check DNA quality and 16S content prior to sequencing, universal primers were used for SYBR Green quantitative polymerase chain reaction (qPCR) with the following extended cycling protocol: 95°C 10min; 95°C 15sec, 60°C 30sec, 72°C 30sec for 40 cycles. Sequencing was completed at the Cincinnati University Children’s Hospital Medical Center’s DNA Sequencing and Genotyping Facility Core (Cincinnati, OH) as described [14]. All antibiotic-treated samples failed to yield 16S rDNA sequence data; one sample each from the ethanol- and pair-fed groups was excluded based on insufficient sequence data.

UPARSE [15] and UTAX (http://www.drive5.com/usearch/manual/cmd_utax.html) were used to generate OTU tables from 16S rDNA read data and to make taxonomic assignments. QIIME package scripts were used for calculations of α- (*PD_whole_tree*, *chao1*, *observed_otus* and *shannon*) and β- (*Bray-Curtis*, *Un-Weighted UniFrac*, and *Weighted UniFrac*) diversity [16].

### Antibiotic treatment and bacterial colony count

Some mice were treated twice daily orally with an antibiotic cocktail containing Ampicillin (100mg/kg BW; Sigma), Neomycin (100mg/kg BW; Gibco), Metronidazole (100mg/kg BW; Sigma) and Vancomycin (50mg/kg BW; Sigma) beginning at the initiation of liquid diet and continuing until the oral ethanol or maltose dextrin gavage on the final feeding day. Non-antibiotic-treated mice received an equivalent volume of water. Gavage needles were rinsed with acid-treated water and sterilized in a hot-bead sterilizer between each mouse treatment to minimize transfer of microbes between animals.

Feces were collected directly from the anus prior to euthanization and frozen at -20°C until plating. Upon thawing, stool was immediately weighed, dissociated in liquid thioglycolate media (Sigma) and diluted 1:1000 prior to plating on non-selective agar plates (EMD Millipore). Plates were incubated at 37°C for 48h. Colonies were quantified using OpenCFU [17] and were normalized to the mass of stool.

### Histology

OilRed O tissue staining on OCT-embedded frozen liver sections was completed and quantification was performed using ImageJ to assess hepatic steatosis. Formalin-fixed paraffin-embedded liver sections were stained with anti-mouse myeloperoxidase antibody (Abcam) and subsequently labeled with streptavidin-biotin immunoenzymatic antigen for detection with 3,3’-diaminobenzidine (DAB) (UltraVision Mouse Tissue Detection System Anti-Mouse HRP/DAB; Lab Vision).

### Biochemical assays

Serum was separated from whole blood and frozen at -80°C until use. Serum alanine and aspartate aminotransferases were measured using a kinetic method [18]. Serum endotoxin was quantified using Pierce LAL Chromogenic assay (ThermoFisher). Serum MCP-1 level was determined by ELISA (BioLegend).

### mRNA analysis

RNA was extracted from liver tissue using RNeasy (Qiagen) according to the manufacturer’s instructions, including on-column DNase digestion (Zymo Research). cDNA was written from 1μg of RNA and then diluted 1:5 in nuclease-free water. SYBR Green (BioRad) real-time qPCR was performed according to the manufacturer’s instructions. The primers used are listed in Table 1 and 18S was used as a housekeeping gene for *2*^*-ddCt*^ method of RNA expression analysis.

**Table 1:** Real-time PCR primers.

Primer	Forward (5’>3’)	Reverse (5’>3’)
*18S*	GTA ACC CGT TGA ACC CCA TT	CCA TCC AAT CGG TAG TAG CG
*E-selectin*	ATG CCT CGC GCT TTC TCT C	GTA GTC CCG CTG ACA GTA TGC
*Ly6g*	TGC GTT GCT CTG GAG ATA GA	CAG AGT AGT GGG GCA GAT GG
*Mpo*	CAT CCA ACC CTT CAT GTT CC	CTG GCG ATT CAG TTT GG
*Tnfα*	GAA GTT CCC AAA TGG CCT CC	GTG AGG GTC TGG GCC ATA GA
*Cxcl1*	ACT GCA CCC AAA CCG AAG TC	TGG GGA CAC CTT TTA GCA TCT T
*Mcp-1*	CAG GTC CCT GTC ATG CTT CT	TCT GGA CCC ATT CCT TCT TG
*Fasn*	GAG GTG GTG ATA GCC GGT AT	TGG GTA ATC CAT AGA GCC CAG
*Ucp1*	AGG CTT CCA GTA CCA TTA GGT	CTG AGT GAG GCA AAG CTG ATT T
*Prdm16*	CCC CAC ATT CCG CTG TGA T	CTC GCA ATC CTT GCA CTC A
*Scd2*	TAC TAC AAG CCC GGC CTC C	CAG CAG TAC CAG GGC ACC A
*Adrp*	CTG TCT ACC AAG CTC TGC TC	CGA TGC TTC TCT TCC ACT CC
*Lcn2*	CCC CAT CTC TGC TCA CTG TC	TTT TTC TGG ACC GCA TTG

The above forward and reverse sequences of primers were used in real-time PCR. Ly6g: Lymphocyte antigen 6 complex locus G6D; Mpo: myeloperoxidase; Tnfα: tumor necrosis factor-α; Cxcl1: C-X-C motif chemokine ligand 1; Mcp-1 monocyte chemoattractant protein 1; Fasn: fatty acid synthase; Ucp1: uncoupling protein 1; Prdm16: PR-domain zinc finger protein 16; Scd2: stearoyl-CoA desaturase 2; Adrp: adipose differentiation-related protein; Lcn2: Lipocalin-2.

## Results

### Alpha- and beta-diversity of cecal microbiome are unaltered by acute-on-chronic alcohol feeding

We applied 16S rDNA sequencing to define and to assess the changes in the cecal microbiota induced by the acute-on-chronic alcohol feeding in mice. The bacterial load was substantially reduced in antibiotic-treated mice and 16S rDNA sequence was not obtained for that cohort. Of the non-antibiotic-treated mice, samples from 7/8 pair-fed and 9/10 alcohol-fed mice were available for analysis. We observed no significant differences (p>0.05) in α-diversity between pair-fed and alcohol-fed groups (Table 2), indicating that the mean species diversity was not affected by acute-on-chronic alcohol.

**Table 2:** α-diversity of cecal bacterial content.

	*PD_whole_tree*		*chao1*		*observed_otus*		*shannon*	
	Average	Error	Average	Error	Average	Error	Average	Error
Pair-fed	6.76	2.35	82.38	36.25	63.86	30.42	2.85	0.69
EtOH-fed	7.37	2.07	87.86	31.59	69.37	25.33	2.62	0.56

Single rarefaction at a depth of 10,000 sequences indicated no significant difference in α-diversity between pair- and ethanol-fed cecal bacterial communities by four different metrics.

Next, we examined β-diversity to assess the degree of dissimilarity of bacterial communities between samples. These analyses determined no significant differences between pair-fed and alcohol-fed mice. Additionally, comparisons within pair-fed and within alcohol-fed conditions revealed that the β-diversity within each group (intra-group diversity) was not statistically different from diversity between the groups (inter-group diversity; data not shown).

### Alcohol induces taxonomic shifts in bacterial communities

**Fig 1:**
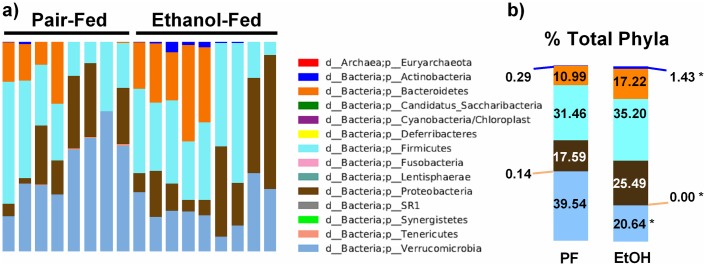
Alcohol induces shifts in various bacterial phyla. (a) Proportional contributions of various bacterial phyla to the overall gut microbiome in pair- and alcohol-fed mice. (b) The average percent of total bacteria measured for each phylum reveals enrichment in *Actinobacteria* (navy blue) in the microbiome of alcohol-fed mice. Phylum *Tenericutes* (beige) was reduced in relative abundance in alcohol-fed mice. The phylum *Verrucomicrobia* (light blue), which represented the majority of bacteria in pair-fed mice, was reduced in the acute-on-chronic ethanol model. The prefix letter followed by an underscore represents the taxonomic level (e.g. “d_” for domain, “p_” for phylum, etc.). * p<0.05 by Mann-Whitney test.

Although α- and β-diversity were not significantly changed, we hypothesized that there may be specific taxonomic shifts after acute-on-chronic alcohol administration between alcohol-fed and pair-fed mice. Compared to pair-fed mice, the phylum *Actinobacteria* was significantly enriched (Fig 1; d_Bacteria;p_Actinobacteria, PF: 0.29% vs EtOH: 1.43%), while phylum *Tenericutes* was reduced in relative abundance in alcohol-fed mice (Fig 1; d_Bacteria;p_Tenericutes, PF: 0.14% vs EtOH: 0.00%). The most abundant phylum, *Verrucomicrobia* (Fig 1; d_Bacteria;p_Verrucomicrobia) showed the greatest reduction in alcohol-fed mice (PF: 39.54% vs EtOH: 20.64%).

**Fig 2:**
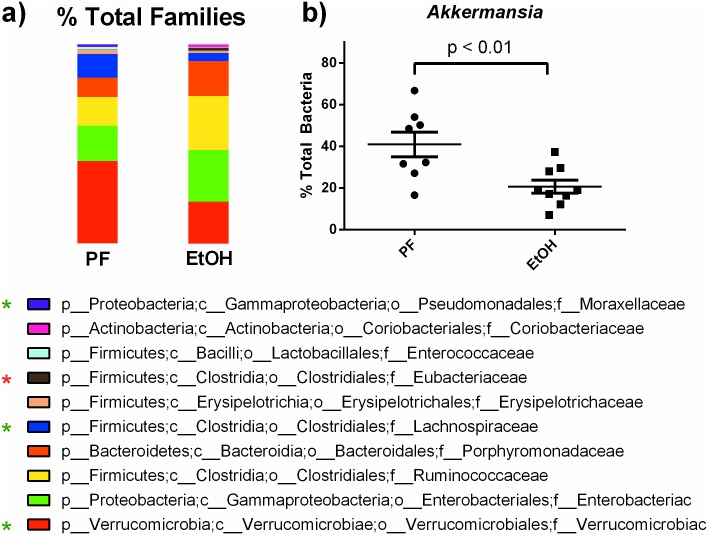
Alcohol alters family-level community representation in some of the nine most abundant bacterial families. (a) To represent a subset of the bacterial composition in cecal content of pair- and alcohol-fed mice, we chose the nine most abundant families, each comprising >0.5% of all families. Those differentially enriched in pair-fed mice are indicated by a green asterisk; red asterisk denotes families enriched in alcohol-fed mice. (b) The genus *Akkermansia* represented 100% of the family *Verrucomicrobiacea*, the most abundant family found in pair-fed mice, and was significantly reduced by alcohol administration. The prefix letter followed by an underscore represents the taxonomic level (e.g. “p_” for phylum, “c_” for class, etc.). * p<0.05 by Mann-Whitney test.

An examination of the nine most abundant families represented in our sample set (Fig 2a) revealed that the most abundant family in cecal content was Verrucomicrobiaceae, which was significantly reduced in alcohol-fed animals when compared to the pair-fed group. Other families with reduced abundance in alcohol-fed mice included Lachnospiraceae and Moraxellaceae (green asterisk), while Eubacteriaceae was enriched in alcohol-fed mice (red asterisk). Examination of the genus composition of the Verrucomicrobiaceae family revealed only one genus, *Akkermansia* which accounted for the reduction observed in alcohol-fed mice from the phylum (Verrucomicrobia) to the genus level (Fig 2b). This analysis identifies reduction of *Akkermansia* as an early marker of alcohol-induced changes in the gut microbiome.

### Antibiotic treatment reduces serum endotoxin and gut bacterial load

**Fig 3:**
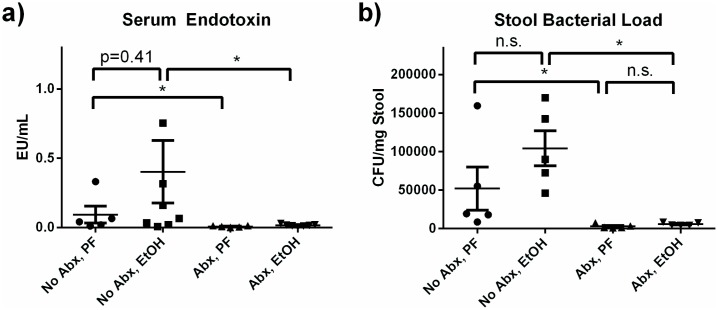
Antibiotics successfully reduce gut bacterial load and circulating endotoxin. (a) Antibiotic treatment reduced the endotoxin levels detected in systemic circulation and abrogated any increase associated with alcohol consumption. (b) Intestinal bacterial load, measured by colony forming units from stool taken directly from the anus prior to sacrifice, was reduced by antibiotics. * p<0.05 by Mann-Whitney test.

Intestinal microbiota and bacterial products are involved in the pathogenesis of alcoholic liver disease [19, 20]. To directly test the impact of gut bacterial load on alcohol-induced inflammation, we first used an antibiotic cocktail to reduce the intestinal bacterial flora. In a preliminary study, we found that mice became averse to drinking water containing the dissolved antibiotic cocktail, which has also been observed by others [21]. We therefore switched to a twice-daily oral gavage regimen to ensure adequate bacterial clearing. Antibiotics were started on the first day of liquid diet acclimatization and continued throughout the alcohol feeding. Antibiotic treatment did not affect daily alcohol diet intake (EtOH-fed: 9.1 ± 0.6 mL/day/mouse; EtOH-fed plus antibiotics: 8.7 ± 0.2 mL/day/mouse; p = 0.14). Serum endotoxin was reduced at the conclusion of the feeding after antibiotic administration (Fig 3a). Fecal samples collected directly from the anus were plated on non-selective agar plates. Significant reduction in the number of colony forming units (CFUs) in antibiotic-treated mice (Fig 3b) confirmed intestinal bacterial decontamination.

### Neutrophil infiltration to the liver is attenuated in mice with reduced gut bacterial load

**Fig 4:**
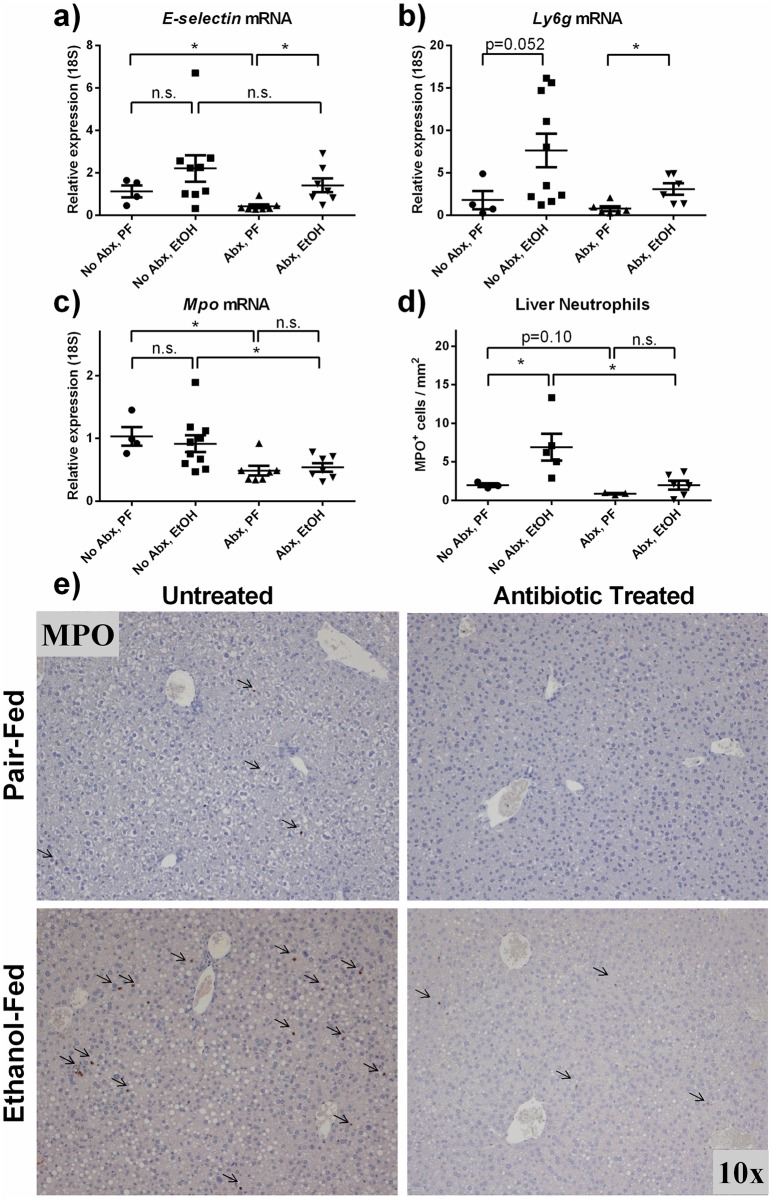
Neutrophil infiltration to the liver is attenuated in mice with reduced gut-bacterial load. (a-c) Liver expression of neutrophil associated genes *E-selectin*, *Ly6g* and *Mpo* were measured by qPCR and normalized to 18S. (e) MPO immunohistochemical staining to detect neutrophils in the liver parenchyma revealed an increased number of neutrophils/area in alcohol-fed mice compared to pair-feds and reduced gut bacterial load led to fewer infiltrating neutrophils. Interestingly, antibiotics also cause a decreased trend in hepatic neutrophils at baseline in pair-fed mice (quantified in (d)). * p<0.05 by Mann-Whitney test.

Having established a reduction in bacterial load and circulating LPS, we hypothesized that this might reduce hepatic inflammation. To test this, we measured liver mRNA expression of the neutrophil associated genes including *E-selectin* (associated with neutrophil attraction), *Ly6g* (a neutrophil-specific marker) and myeloperoxidase (*Mpo*; an enzyme involved in processing phagocytic material in neutrophils). *E-selectin* was not significantly increased by alcohol, although antibiotics reduced its expression and alcohol-fed antibiotic-treated mice did display an increase when compared to pair-fed plus antibiotics. The trend toward increased *Ly6g* expression following alcohol administration (p = 0.052) was not abrogated after gut decontamination by antibiotics in alcohol-fed mice. Liver *Mpo* mRNA expression was reduced by antibiotics in both control and alcohol-fed mice (Fig 4a–4c). The increased involvement of neutrophils in the liver following alcohol consumption was confirmed by MPO immunohistochemical staining. Pair-fed mice treated with antibiotics had fewer neutrophils at baseline (p = 0.10) and antibiotic-treated mice were protected from alcohol-induced increase in infiltrating neutrophils in the liver (Fig 4d and 4e).

### Gut decontamination reduces alcohol-induced inflammatory mediators in the liver and circulation

**Fig 5:**
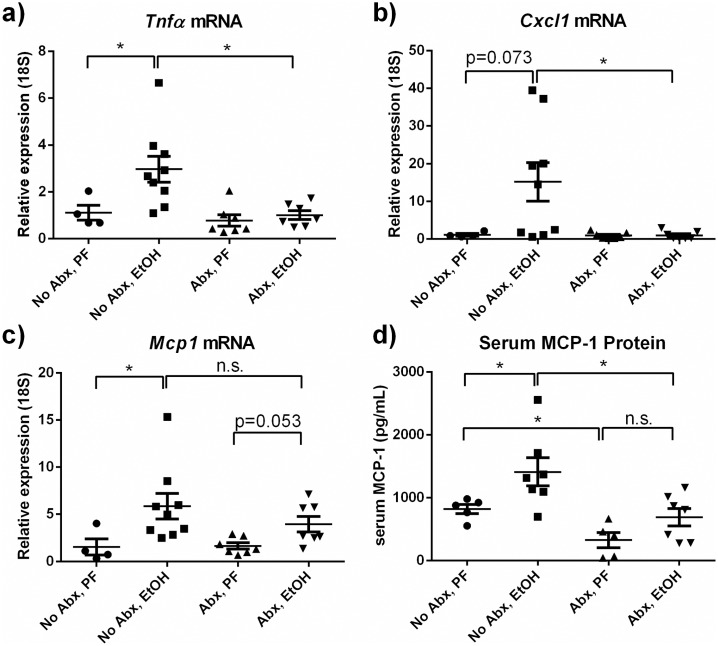
Antibiotic treatment reduces expression of inflammatory mediators in the liver and circulation. (a-b) Liver expression of the cytokine *Tnfα* and the chemokine *Cxcl-1* is increased by alcohol and antibiotics eliminate these increases. (c) Although mRNA levels of *Mcp-1* are not different between the antibiotic-treated/untreated alcohol-fed groups, (d) circulating MCP-1 protein is reduced in alcohol-fed antibiotic-treated mice. * p<0.05 by Mann-Whitney test.

Liver inflammation is a major component of the pathomechanism of alcoholic hepatitis in humans and in animal models. To test if liver inflammation is mediated by bacterial load, we measured transcripts of key cytokines and chemokines from liver tissues by qPCR. Expression of *Tnfα*, *Cxcl1* and *Mcp-1* mRNA was elevated in alcohol-fed mice compared to pair-fed controls (p<0.05 for *Tnfα*, *Mcp-1*; p<0.1 for *Cxcl1*). Importantly, increases in *Tnfα* and *Cxcl1* were eliminated by antibiotic treatment in alcohol-fed mice (Fig 5a–5c). Circulating MCP-1 protein from serum showed an alcohol-induced increase that was mitigated by antibiotic reduction of intestinal bacteria suggesting that gut decontamination has an important effect on liver inflammation in alcoholic hepatitis (Fig 5d). Together, this data showed that treatment with antibiotics reduced circulation of MCP-1 and expression of inflammatory markers.

### Suppressed bacterial load reduces alcohol-induced liver steatosis but not serum transaminases

**Fig 6:**
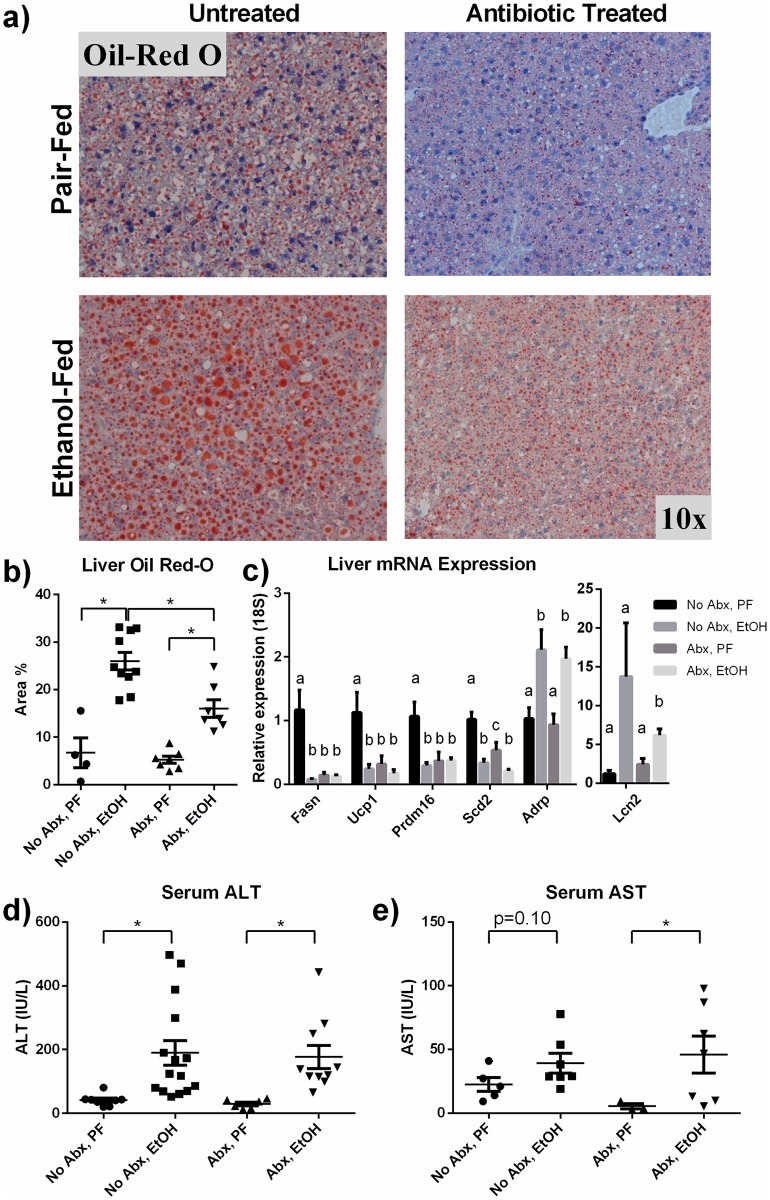
Steatosis but not serum transaminase increase from alcohol consumption is reduced by gut sterilization. (a-b) Oil-red O staining on liver sections reveals that alcohol causes a dramatic increase in hepatic steatosis. (c) Liver mRNA expression of various genes involved in lipid metabolism, including fatty acid synthase (*Fasn*), uncoupling protein-1 (*Ucp1*), PR-domain zinc finger protein 16 (*Prdm16*), stearoyl-CoA desaturase 2 (*Scd2*), adipose differentiation-related protein (*Ardp*) and lipocalin-2 (*Lcn2*) were measured by qPCR. (d) Serum alanine (ALT) and (e) aspartate (AST) transaminases, which are increased by acute-on-chronic alcohol, are unchanged by antibiotic treatment. For (c), groups that do not share a letter are significantly different; * p<0.05 by Mann-Whitney test.

Antibiotic treatment resulted in pro-inflammatory signal reduction, suggesting that perhaps other aspects of alcoholic liver disease, such as hepatic steatosis, may also be impacted. To test this, we used Oil Red-O staining and observed that alcohol-fed mice had increased hepatic steatosis compared to pair-fed mice. However antibiotic treatment reduced the alcohol-related increase in steatosis (Fig 6a and 6b). To investigate the effect of alcohol and antibiotic treatment on lipid regulation and synthesis, we measured mRNA levels of various liver genes in lipid metabolism (Fig 6c). Alcohol feeding reduced expression of fatty acid synthase (*Fasn*), uncoupling protein-1 (*Ucp1*), PR-domain zinc finger protein 16 (*Prdm16*), stearoyl-CoA desaturase 2 (*Scd2*). Antibiotic treatment, irrespective of alcohol feeding, also reduced expression of *Fasn*, *Ucp1*, and *Prdm16* while antibiotics plus ethanol further reduced *Scd2* compared to antibiotics plus pair-fed. Adipose differentiation-related protein (*Ardp*) is a molecule that coats intracellular stores of lipid and lipocalin-2 (*Lcn2*) is involved in intracellular lipid transport. *Adrp* expression was elevated in alcohol-fed animals regardless of antibiotic treatment and *Lcn2* trended toward an increase (p = 0.10) in alcohol-fed animals and was significantly increased in alcohol plus antibiotic mice, reflecting the increase in liver Oil Red-O in alcohol-fed animals. Serum alanine (ALT; Fig 6d) and aspartate (AST; Fig 6e) aminotransferases were elevated in mice fed alcohol compared to those receiving control diet, indicating hepatocyte injury. Interestingly, serum ALT levels remained elevated in ten days plus binge alcohol-fed mice receiving antibiotic treatment, suggesting microbe-independent mechanisms of alcohol-induced hepatocyte injury.

## Discussion

This study provides, for the first time, insight into the microbiome changes that occur in the mouse model of early acute alcoholic steatohepatitis. The model does not induce differences in the α-diversity of or the β-diversity between intestinal bacterial communities in alcohol and pair-fed mice. However specific taxonomic shifts did reflect important changes in the makeup of the bacterial community in alcohol-fed mice and represent early indicators of developing alcoholic liver disease. Specifically, we identify *Akkermansia* decrease as an early change in alcohol-induced intestinal dysbiosis. Our data also reveal the influence of gut bacterial load on liver neutrophil infiltration, inflammation signaling and steatotic changes.

Our study utilized the acute-on-chronic feeding which spans a total of ten days. Others have investigated microbiome shifts in long-term alcohol feeding paradigms and observed α- and β-diversity changes at later time points. In one such study [22], diversity measurements were increased in alcohol-fed mice after six-weeks of alcohol, while earlier time points were not investigated. That same study also showed trends in stool samples similar to ours, including a modest increase in the phylum Actinobacteria after alcohol administration. These similarities are particularly interesting because that study analyzed bacterial content of the stool while we examined bacteria from cecal content. In a short alcohol exposure model using seven day intragastric alcohol infusion, Wang et al. described an increase in β-diversity between alcohol-infused and control mice [23]. However, the intragastric infusion model varies significantly from the orally consumed Lieber-DeCarli liquid diet used in our study where the consumption method alone could explain the discrepant diversity findings.

In analysis of cecal content, we observed various taxonomic changes between alcohol- and pair-fed mice. Among these changes, phylum Verrucomicrobia was dominated by *Akkermansia* and its abundance was dramatically reduced by alcohol consumption. This finding is corroborated by careful investigation of other studies [23] and offers a promising look into the potential importance of this genus in gut barrier maintenance disrupted by alcohol. Indeed, *Akkermansia* has been found to be decreased in mouse models of obesity and type-2 diabetes, while supplementation with the bacteria alleviated the burden of metabolic dysfunction [24].

Beyond the effects of alcohol on cecal bacterial community changes, we also investigated the role of overall bacterial load on hallmarks of steatohepatitis showing that gut bacterial load reduction attenuated immune cell infiltration, steatotic accumulation and inflammation in the liver. Infiltration of immune cells, especially neutrophils, is a key component of the acute-on-chronic model in the alcohol field [13, 25]. However, the question of whether neutrophils are brought in to repair damage or whether their presence causes damage in the liver remains important. Here, we reduced the hepatic infiltration of neutrophils by treating with antibiotics without protecting the liver from damage (indicated by increased serum ALT/AST transaminases). This is partially corroborated by Bertola et al. who used an anti-neutrophil (anti-Ly6G) antibody and showed near complete elimination of neutrophil infiltration into the liver but only partial protection from transaminase increase [13]. A possible explanation for the continued elevation of ALT and AST despite protecting the liver from infiltrating inflammatory cells such as neutrophils comes from the nature of the feeding model. This acute-on-chronic model includes an oral gavage of high-dose ethanol after ten days of feeding. The metabolism of this large dose of alcohol in a short time may induce hepatocyte damage and transaminase release regardless of the presence or absence of other damage or pathogen associated signals. Suppression of circulating bacterial components (such as endotoxin/LPS) reduces the inflammatory signaling in the liver that would normally attract immune cells such as neutrophils to the site of liver injury.

We describe the protective role of antibiotic-mediated reduction in bacterial load in alcohol-induced inflammatory signaling, steatotic changes and neutrophil infiltration in the liver. Reducing circulating bacterial products, such as endotoxin (Fig 3b), likely reduces signaling via TLR-4 and subsequent inflammatory signaling [8, 9]. Additionally, the reduction in circulating MCP-1 protein in antibiotic-treated mice may contribute to the protection from alcohol-induced steatosis we report here, as we have previously shown the role of MCP-1 in promoting lipid accumulation [26].

To further investigate the effect of alcohol and bacterial reduction on steatosis, we measured mRNA levels of liver transcripts related to fat metabolism and lipid regulation. Interestingly, we found that ethanol reduced expression of fatty acid synthase (*Fasn*), uncoupling protein-1 (*Ucp1*), PR-domain zinc finger protein 16 (*Prdm16*), stearoyl-CoA desaturase 2 (*Scd2*). Antibiotic treatment also reduced expression of these genes compared to pair-fed mice without antibiotics. Gene suppression by antibiotics is consistent with other studies that have shown antibiotic treatment can have a negative impact on steatotic accumulations in the liver [27].

Adipose differentiation-related protein (*Ardp*) and lipocalin-2 (*Lcn2*) are both involved in storage and transport of accumulated lipids within the cell. Their increased expression (*Lcn2* p = 0.10) in alcohol-fed animals compared with pair-feds, irrespective of antibiotic treatment, mirrors the increased lipid store within cells observed on histology. Therefore, we have observed that genes regulating lipid production tend to be suppressed by antibiotics and alcohol in this feeding model, while genes associated with regulating lipid stores (*Adrp*, *Lcn2*) are elevated in order to compensate for the ethanol-induced fat accumulation.

Many of these findings intriguingly contradict those observed in germ-free mice [28] which displayed significantly greater liver injury, inflammation and steatosis when compared with conventional mice in a model of acute alcohol exposure. In that study, germ free mice were given a single gavage of 3g/kg ethanol and sacrificed 9h later (compared to our treatment of ten days of 5% EtOH in liquid diet and a final gavage of 5 g/kg sacrificed 9h later). This lower ethanol dose induced only modest changes in serum ALT, steatosis and inflammatory cytokine expression in alcohol- versus pair-fed conventional (bacteria-competent) mice. Additionally, while we successfully reduced gut bacterial load using antibiotic treatment, some intestinal microbes still persisted (Fig 3a). It is possible that the remaining bacterial composition contained protective species (although we could not sequence these populations due to the low yield of bacterial DNA) or that the remaining bacteria may serve a homeostatic function in either intestinal metabolism or barrier maintenance [29].

In our study, we observe changes in the liver, including inflammatory cytokine expression, elevated markers of liver injury and immune cell infiltration in the alcohol-fed mice, despite relatively stable communities of gut bacteria (i.e. no dramatic shifts in diversity) compared to long-term alcohol feeding [22]. This suggests that while some features of alcoholic liver disease may be related to shifts in the gut bacterial composition seen over the long-term, there are other features, such as inflammation signaling and immune cell infiltration and hepatocyte injury that may be independent of bacterial diversity changes. Here, we shed critical light on the role of the intestinal microbiome in early alcoholic steatohepatitis and offer new avenues of focus for future research into the gut-liver axis.

## Conclusion

This study supports the hypothesis that the gut microbiota are impacted by alcohol consumption and that the acute-on-chronic feeding model alters the microflora at multiple taxonomic levels. We conclude that gut microbes influence liver inflammation, neutrophil infiltration and liver steatosis following alcohol consumption and these data further emphasize the gut-liver axis, even in early alcohol-induced inflammation.
