# Correction: Alcohol-related changes in the intestinal microbiome influence neutrophil infiltration, inflammation and steatosis in early alcoholic hepatitis in mice

**DOI:** 10.1371/journal.pone.0179070

**Published:** 2017-05-31

**Authors:** Patrick P. Lowe, Benedek Gyongyosi, Abhishek Satishchandran, Arvin Iracheta-Vellve, Aditya Ambade, Yeonhee Cho, Karen Kodys, Donna Catalano, Doyle V. Ward, Gyongyi Szabo

Yeonhee Cho should be included in the author byline instead of the Acknowledgments. She should be listed as the sixth author, and her affiliation is 1: Department of Medicine, University of Massachusetts Medical School, Worcester, Massachusetts, United States of America. The contributions of this author are as follows: Performed the experiments.

The correct citation is: Lowe PP, Gyongyosi B, Satishchandran A, Iracheta-Vellve A, Ambade A, Cho Y, et al. (2017) Alcohol-related changes in the intestinal microbiome influence neutrophil infiltration, inflammation and steatosis in early alcoholic hepatitis in mice. PLoS ONE 12(3): e0174544. https://doi.org/10.1371/journal.pone.0174544
